# Consistent mapping of government malaria records across a changing territory delimitation

**DOI:** 10.1186/1475-2875-13-S1-P5

**Published:** 2014-09-22

**Authors:** Ricardo Andrade-Pacheco, Martin Mubangizi, John Quinn, Neil D Lawrence

**Affiliations:** 1 Department of Computer Science, University of Sheffield, Sheffield, UK; 2 Department of Computer Science, Makerere University, Kampala, Uganda

## Background

Health Management Information Systems (HMIS) are a crucial tool for supporting planning and decision-making. The benefits of such systems will depend on the quality of the data they provide and on the response capacity of the decision-makers [1]. The analysis of malaria incidence records of the HMIS, in Uganda, faces two main complications. First, artificial trends induced by a non-negligible and variable rate of non-reporting hospitals. Second, lack of comparability across time, due to changes in the districts boundaries.

## Materials and methods

We propose a method for estimating the incidence of malaria for the different district definitions across time. Although we have information for the whole country across many years, this task requires making estimates for periods where data is not available for a specific district delimitation. We provide disease maps based on HMIS information by exploiting the relationship with its environmental drivers. Our approach relies on the Gaussian process framework. In particular, we use multiple output kernel techniques [2] to achieve consistency between the totals and subtotals of incidence records at different levels of territory aggregations. In the case of map generation, this approach allows us to combine information from different sources at different spatial resolution. We use the HMIS malaria records from 2003 to 2013. The records consist of weekly information aggregated within districts. The information available also includes the number of hospitals reporting each week. We use the normalized difference vegetation index and land surface temperature measurements, both commonly used for identifying suitable habitats for mosquito breeding [3].

## Results

For recently created districts, our method allows comparability between the current malaria incidence and periods before they started reporting to the HMIS. The probabilistic model defined allows HMIS users to generate samples from a incidence distribution to develop further analysis. We also generate disease maps by combining administrative records with remote sensing data.
